# Widespread and active piezotolerant microorganisms mediate phenolic compound degradation under high hydrostatic pressure in hadal trenches

**DOI:** 10.1007/s42995-024-00224-2

**Published:** 2024-05-13

**Authors:** Hao Ling, Yongxin Lv, Yu Zhang, Ning-Yi Zhou, Ying Xu

**Affiliations:** 1 State Key Laboratory of Microbial Metabolism, Joint International Research Laboratory of Metabolic and Developmental Sciences and School of Life Sciences and Biotechnology, Shanghai Jiao Tong University, Shanghai, 200240 China; 2 School of Oceanography, Shanghai Jiao Tong University, Shanghai, 200240 China; 3 State Key of Laboratory of Ocean Engineering, Shanghai Jiao Tong University, Shanghai, 200240 China

**Keywords:** Hadal trench, High hydrostatic pressure, Phenolic compounds degradation, Piezotolerant microorganisms, Widespread distribution

## Abstract

Phenolic compounds, as well as other aromatic compounds, have been reported to be abundant in hadal trenches. Although high-throughput sequencing studies have hinted at the potential of hadal microbes to degrade these compounds, direct microbiological, genetic and biochemical evidence under in situ pressures remain absent. Here, a microbial consortium and a pure culture of *Pseudomonas*, newly isolated from Mariana Trench sediments, efficiently degraded phenol under pressures up to 70 and 60 MPa, respectively, with concomitant increase in biomass. By analyzing a high-pressure (70 MPa) culture metatranscriptome, not only was the entire range of metabolic processes under high pressure generated, but also genes encoding complete phenol degradation via *ortho*- and *meta*-cleavage pathways were revealed. The isolate of *Pseudomonas* also contained genes encoding the complete degradation pathway. Six transcribed genes (*dmpKLMNOP*_*sed*_) were functionally identified to encode a multicomponent hydroxylase catalyzing the hydroxylation of phenol and its methylated derivatives by heterogeneous expression. In addition, key catabolic genes identified in the metatranscriptome of the high-pressure cultures and genomes of bacterial isolates were found to be all widely distributed in 22 published hadal microbial metagenomes. At microbiological, genetic, bioinformatics, and biochemical levels, this study found that microorganisms widely found in hadal trenches were able to effectively drive phenolic compound degradation under high hydrostatic pressures. This information will bridge a knowledge gap concerning the microbial aromatics degradation within hadal trenches.

**Supplementary Information:**

The online version contains supplementary material available at 10.1007/s42995-024-00224-2.

## Introduction

Hadal zones (≥ 6000 m below sea level; mbs) are dominated by deep trenches formed along tectonic plate boundaries by the convergent movements between oceanic and terrestrial plates (Nunoura et al. 2015). Due to oceanic hydrodynamic activity and a funneling effect resulting from their “V” shaped morphology (Ichino et al. 2015; Itou et al. 2000), hadal trenches receive a diverse range of organic matter from overlying water columns, and, therefore, serve as a carbon sink in global oceans (Shigemitsu et al. 2021; Xu et al. 2018; Zhang et al. 2022). In particular, aromatic compounds from both natural and anthropogenic sources have been reported to be present at the trench bottoms (Cui et al. 2020; Dasgupta et al. 2018; Jamieson et al. 2017).

Despite the harsh conditions, such as extremely high hydrostatic pressure (≥ 60 MPa) (Jamieson et al. 2010; Nunoura et al. 2015), distinctive and thriving hadal microbial communities, especially heterotrophic prokaryotes, have been well documented at these depths (Liu et al. 2020; Nunoura et al. 2018; Tarn et al. 2016; Tian et al. 2018). The carbon turnover observed at the trench bottom has been found to be more intense than in the adjacent abyssal plains, confirming the active role of indigenous piezophilic heterotrophs in driving hadal biogeochemical cycling (Glud et al. 2013; Wenzhöfer et al. 2016). Much progress has been made in characterizing the microbial structure and versatile metabolic functions in these regions (Chen et al. 2021; Zhang et al. 2018; Zhou et al. 2022). Furthermore, many diverse piezophilic bacterial strains have been isolated from trench sediments using nutrient-rich medium (Pathom-Aree et al. 2006a; Tamegai et al. 1997; Yan et al. 2020; Yang et al. 2020), and several strains were found that had the ability to utilize alkanes in simulated hadal environments (Liu et al. 2019). Aromatic compounds, in addition to being a significant deep ocean carbon source, are potential carbon sources for hadal ecosystems (Liu et al. 2022). Based on metagenomic sequencing and bioinformatics analysis, genes possibly involved in monocyclic aromatic compound degradation have been identified from hadal microbial metagenomes (Chen et al. 2021; Wei et al. 2020; Xue et al. 2020; Zhang et al. 2018). In particular, the gene encoding a putative catechol 2,3-dioxygenase, an aromatic ring-fission enzyme, was reported to be highly transcribed in three hadal SAR202 metagenome-assembled genomes (Gao et al. 2019). Moreover, several putative catabolic genes responsible for the degradation of polyaromatic hydrocarbons and polychlorinated biphenyls were found to be present in *Chloroflexi* metagenome-assembled genomes from trench sediment (Liu et al. 2022). These cultivation-independent studies implied the presence of potential aromatic utilizers inhabiting hadal trenches, which would play a crucial role in recycling the relatively recalcitrant ring structure of these compounds. However, the cultivation of an aromatic-degrading microbial consortium or pure isolates from hadal trenches has yet to be achieved. Furthermore, it is unclear whether the mechanisms of microbial-driven carbon cycling of aromatic compounds are similar in different global trench environments. Thus, further evidence of cultivation is required to support the hypothesis that trench-derived microbes degrade aromatic compounds under high hydrostatic pressure (Zhou et al. 2022) as well as understanding the related processes at the molecular and enzymatic levels.

Phenolic compounds, monocyclic aromatic compounds originating from hydrothermal petroleum as well as the natural decomposition of phytodetritus, microbial metabolites and industrial sewage release (Duan et al. 2018; Lochab et al. 2014; Saito et al. 2018; Simoneit 2018), are frequently found in marine and freshwater systems (Anku et al. 2017; González-Gaya et al. 2019). These compounds tend to accumulate with increasing depth from 0 to 2000 m, according to an investigation of the upper 2000 m in Mariana Trench (Li et al. 2019). This study reports phenolic compound degradation by a newly enriched hadal microbial consortium under hadal environment-like high-pressure conditions, the function of catabolic genes encoding phenolic compounds degradation via diverse degradation pathways and the distribution and abundance of key catabolic genes in several major microbial taxa across three different hadal trenches. Moreover, a piezotolerant phenol degrader containing a phenol catabolic cluster was also isolated from trench sediments. This study fills a gap in our understanding of microbial aromatic degradation under high hydrostatic pressures at the microbial, biochemical, and molecular levels, and illuminates their potential roles in carbon cycling within hadal environments.

## Materials and methods

### Chemicals, strains, plasmids, and media

Phenol, *ortho*-, *meta*-, and *para*-cresol, 3-methyl and 4-methylcatechol (purity grade ≥ 99% for all) were all purchased from Sigma Chemical Company (St. Louis, MO, USA). Strains, plasmids, and primers are listed in Table 1. *E*. *coli* strains were grown in lysogeny broth (LB) (Bertani 1951, 2004) at 37 °C. Hadal trench-derived microbes were grown in basal artificial seawater medium (ASW) (Ley et al. 2023; Widdel and Bak 1992) with phenol during high-pressure incubation.

**Table 1:** Bacterial strains, plasmids, and primers used in this study

Bacterial strain, plasmid, or primer	Description or sequence^a^ (5′–3′)	Source or reference
*Bacterial strains*		
*E. coli* BL21(DE3)	F^−^ *ompT hsdS*_*B*_ (rB^−^mB^−^) *gal* (*λcl857 ind1 Sam7 nin5 lac*UV5-T7gene1) *dcm* (DE3)	Novagen
*Pseudomonas* sp. strain NyZ704	Wild type, capable of growing on phenol under 60 MPa high pressure	This study
*Plasmids*		
pET-28a(+)	Km^r^, overexpression vector	Novagen
pMD-18T	Ap^r^, cloning vector	Takara
*Primers*		
519f	CAGCMGCCGCGGTAA	Universal forward primer for archaeal 16S rRNA gene fragment
908r	CCCGCCAATTCCTTTAAGTT	Universal reverse primer for archaeal 16S rRNA gene fragment
341f	CCTACGGGWGGCWGCA	Universal forward primer for bacterial 16S rRNA gene fragment
518r	TTACCGCGGCKGCTG	Universal reverse primer for bacterial 16S rRNA gene fragment
*dmp*_F	*CAGCAAATGGGTCGCGGATCC*TGATTTCTTATTGTGGTTTTGAGTGTT	Forward primer for *dmpKLMNOP*_*sed*_
*dmp*_R	*GCAAGCTTGTCGACGGAGCTC*ACCGCTTAGATGCGCTTGAA	Reverse primer for *dmpKLMNOP*_*sed*_

^a^The homologous arms of pET-28a( +) are underlined and italicized

### Sample information and high-pressure incubation

Trench sediments were collected from the Challenger Deep in the Mariana Trench at depths of 6300 mbs (142.2260° E, 10.8895° N) and 8636 mbs (141°48.7008′ E, 11°11.6988′ N). The sediments were sealed in sterile bags in the dark at 4 °C under ambient pressure for about 2 months until use. For high-pressure incubation of different aims, the sediment sample or pure bacterial isolate was inoculated in basal ASW with the addition of differing amounts of phenol. The culture was injected into sterile syringes with rubber seals immediately followed by pressurizing to specific pressures using high-pressure bottles (developed at Shanghai Jiao Tong University, China). The cultures were then incubated at temperatures of 10–15 °C. Sampling was conducted at appropriate intervals, with 2 mL samples removed from the initial syringe and the remaining culture re-pressurized. After centrifugation at 12,000×*g* at 4 °C for 10 min, the supernatants of samples were used for HPLC analysis, and the cells were used for biomass quantification.

### Biomass quantification

The total DNA of cells obtained by centrifugation was extracted using a FastDNA spin kit for soil (MP Biomedicals, California, USA), according to the manufacturer’s instructions. The 16S rRNA gene fragments were amplified for biomass quantification with a CFX96 real-time PCR detection system (Bio-Rad, USA) using TB Green Premix Ex Taq II (Tli RnaseH Plus) (2X) (Takara, USA). Primers 341f and 518r were used for bacteria, and primers 519f and 908r were used for archaea (Table 1). The amplified bacterial or archaeal 16S rRNA gene fragments were purified and cloned into pMD-18T. Then, the recombinant plasmids were used as the templates for real-time PCR with the aforementioned pairs of primers. A negative control was used to monitor potential contamination, and melting curves were monitored to confirm the absence of nonspecific amplification. All of the experiments in this study were performed in triplicate.

### Metatranscriptomics RNA extraction and shotgun sequencing

The enriched culture with phenol was immediately centrifuged at 12,000×*g* at 4 °C for 10 min after depressurization, and the precipitate was used for RNA extraction and transcriptome sequencing. Total RNA was extracted using an RNA PowerSoil® total RNA isolation kit (12866-25) (MoBio, USA), according to the manufacturer’s instructions. The extracted RNA was assayed using 1.5% agarose gel electrophoresis and a UV spectrophotometer for quality determination and quantification. RNA integrity (RIN ≥ 5.5) was measured using an Agilent 2100 (Agilent, USA). After the removal of ribosomal RNA, Illumina’s TruSeq Stranded mRNA LT sample prep kit (Illumina, USA) was used for reverse transcription as well as macro-transcriptome birdshot sequencing library construction. Each library was sequenced by an Illumina NovaSeq platform (Illumina, USA) using the PE150 strategy at Personal Biotechnology Co., Ltd. (Shanghai, China).

### Metatranscriptomics analysis

Raw sequencing reads were processed to obtain quality-filtered reads for further analysis. First, sequencing adapters were removed from sequencing reads using Cutadapt (v1.17) (Martin 2011). Next, low quality reads were trimmed using a sliding window algorithm in fastp (v0.20.0) (Chen et al. 2018). Then, ribosomal RNA was removed by SortMeRNA (v4.2.0) (Kopylova et al. 2012) using its default rRNA reference database. Once quality-filtered reads were obtained, taxonomical classifications of metatranscriptomics sequencing reads from each sample were performed using Kraken2 (Wood et al. 2019) against a RefSeq-derived database. Cleaned reads were de novo assembled using Trinity software (version: trinityrnaseq2.0.6) with default parameters (Haas et al. 2013). The transcriptomic genes were then predicted from contigs using TransGeneScan software (Ismail et al. 2014) and functionally annotated with Diamond (Buchfink et al. 2015) against multiple databases. The transcriptional level of the genes was evaluated by transcripts per million (TPM), which is a normalization method based on gene length and the corresponding mapped reads number. KO was obtained using KOBAS (Bu et al. 2021). For the local blast program, the BLAST + tool kit (v2.13.0) was downloaded from the NCBI website as https://ftp.ncbi.nlm.nih.gov/blast/executables/blast+/LATEST/.

### Molecular cloning, heterologous expression, and biotransformation

The *dmp* gene cluster derived from metatranscriptome in this study was synthesized de novo by Tsingke Biotechnology Co., Ltd. (Beijing, China). Then it was cloned into pET-28a(+) (Novagen, USA) and expressed in *E. coli* BL21(DE3). The cells were cultivated in LB at 37 °C to an OD_600_ of 0.6 and then were induced at 16 °C for 16 h with 0.2 mmol/L isopropyl β-D-1-thiogalactopyranoside. The cells were then harvested and washed twice with phosphate-buffered saline (pH 7.4). After resuspending the cells with phosphate-buffered saline to an OD_600_ of 10, biotransformation was conducted at 37 °C against phenol and its three methylated derivatives. At appropriate intervals, 2 mL samples were withdrawn and centrifuged. After centrifugation at 12,000×*g* and 4 °C for 10 min, the supernatants were used for HPLC or GC–MS analysis. Phenol and its derivatives were detected by HPLC, and the products were analyzed using GC–MS. For GC–MS analysis, the supernatants were extracted with two volumes of sodium hydroxide-washed ethyl acetate. The fractions containing the products were evaporated to dryness. The dried samples were dissolved in ethyl acetate and added to an equal volume of N,O-bis (trimethylsilyl) trifluoroacetamide (BSTFA) for TMS derivatization at 75 °C for 35 min before analysis as described previously (Li et al. 2023). All of the proposed products were identified by comparing retention times and mass spectra with those of authentic standards.

### Chemical analysis

HPLC analysis was performed on a Waters e2695 separation module with a 2998 PDA detector. An Agilent ZORBAX SB-C18 column (5 μm, 4.6 mm × 250 mm) was used to separate aromatic compounds. The mobile phase containing 70% distilled water acidized with 0.1% (v/v) acetic acid and 30% acetonitrile was pumped isocratically at a flow rate of 1 mL/min at 30 °C for 20 min. The eluent was monitored at 270 nm. GC–MS analyses were conducted on a TRACE 1310 gas chromatograph (Thermo Fisher Scientific, USA) using a capillary column HP-5MS (0.25 mm × 30 m, Agilent Technologies, USA) with methods described previously (Li et al. 2023).

### Metagenomic analysis and taxonomic annotation

The 22 publicly available oceanic trench metagenomes were downloaded from the NCBI SRA database under the accession numbers SRP119520, SRR6057435, SRR6057749, SRR7974510, SRR6057436, SRX11046956, SRX11046361, SRX11044740, and SRP15190, which were derived from the Mariana Trench, Yap Trench, and Kermadec Trench in the Pacific Ocean, covering water depths from 0 to 10,500 mbs, and including seawater (free-living and particle-associated) and sediment samples. The 150-bp paired-end raw reads were first trimmed by BBDuk tools (https://sourceforge.net/projects/bbmap/) with a sequence quality score of 20 and a final minimum length of 90 bp. The DNA reads were assembled using megahit with the following parameters: –min-count 2 –m 1 –k-step 4 –k-min 21 –k-max 141 –cleaning-rounds 10. The assembly was filtered for a minimum length of 500 bp using a custom Python script. Then, reads from each incubation were mapped to the filtered assembly separately by BBMap with *k* = 13 minid = 0.95 pairlen = 350 rescccuedist = 650. The mapped file in SAM format was converted to BAM format and sorted by SAMtools (Li et al. 2009). Genes were predicted by Prodigal (Hyatt et al. 2010) for the filtered assembly and those with a length less than 100 bp were discarded. The modified gene set was functionally annotated with an integrated result, with the following priorities: GhostKOALA (Kanehisa et al. 2016), emapper (version 2.0.1) against the EggNOG database (version 5), and KofamKOALA (version 1.0.3). FeatureCounts (Liao et al. 2014) was used to count the read number of each gene, and the TPM value was calculated with a custom Python script. The query sequences of targeted proteins were subjected to BLASTp alignment against metagenomic proteins using DIAMOND (v0.9.25.126) with an e value threshold of 1 e^−5^ and identity and coverage of 30%. Taxonomy was determined by aligning genes against the representative genes of the GTDB database (version 202) by DIAMOND.

### Isolation of the phenol degrader from trench sediments

Two Mariana Trench sediment samples from 6,300 mbs and 8,636 mbs were individually incubated with phenol in basal ASW under 60 MPa from 10 to 15 °C for 20 days. Then, two enrichments were diluted serially and spread on LB agar plates for incubation at 16 °C for 14 days. The culturable colonies were further incubated with phenol in liquid basal ASW at 16 °C under ambient pressure for isolation of the phenol degrader.

### Phylogenetic analysis and conserved amino acid sequence analysis

Multiple sequence alignment was conducted using the MUSCLE program of MEGA11 software. The resulting alignment file was then used for constructing a maximum-likelihood phylogenetic tree using the IQ-TREE (version 2.0.3) program with the following settings: -B 1000 -m TEST. The consensus tree was then visualized using the interactive Tree Of Life (iTOL v.5) tool (Letunic and Bork 2021). Conservation of amino acids at specific locations in protein sequences was visualized using WebLogo3 (http://weblogo.threeplusone.com/) (Crooks et al. 2004; Schneider and Stephens 1990).

## Results

### Trench-derived microbes drove phenol degradation with concomitant increases in biomass in a simulated hadal environment

**Fig. 1:**
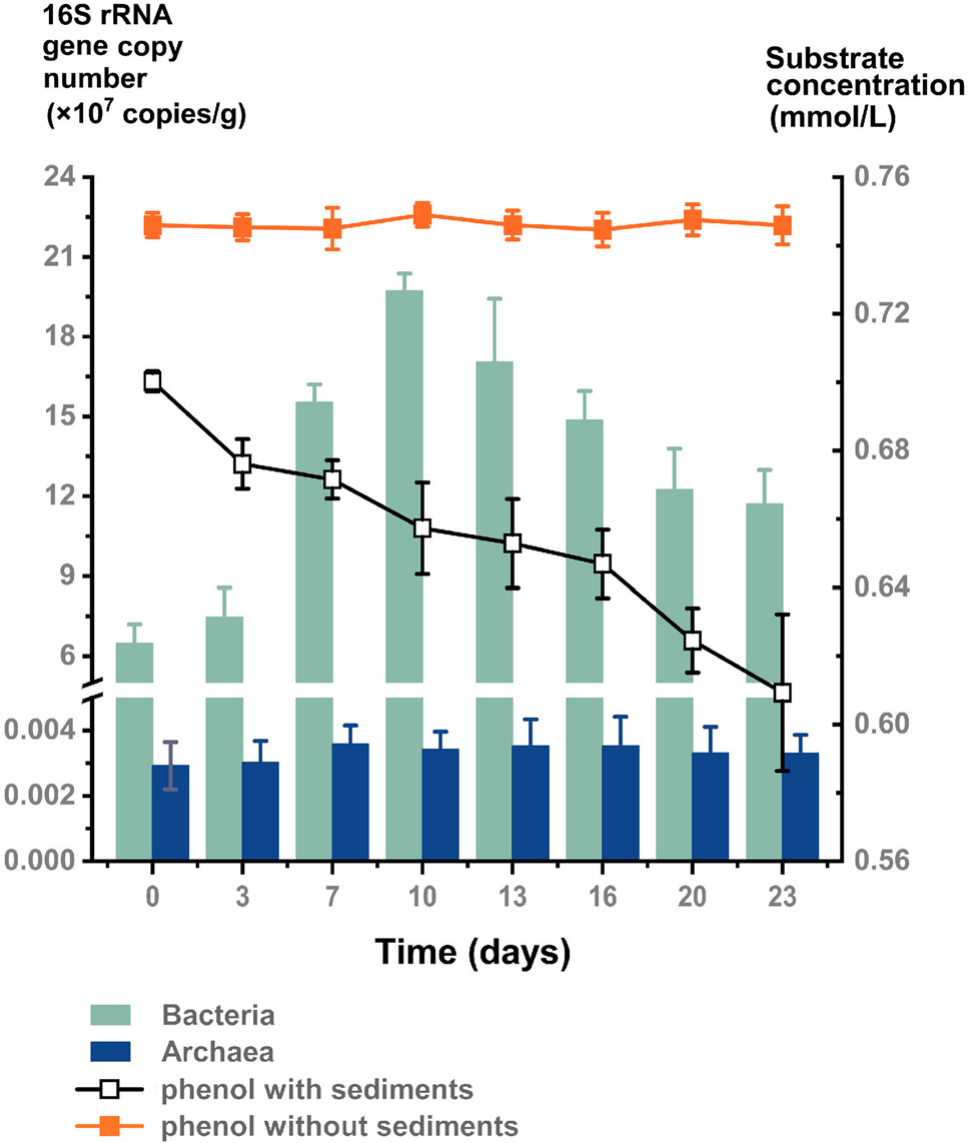
Changes in microbial biomass and phenol concentration during high-pressure incubation at 70 MPa. The 16S rRNA gene copy number and substrate concentration represent the mean values from triplicate experiments, with error bars denoting standard deviations

To test the ability of trench microbes to utilize phenolic compounds in a hadal environment, Mariana Trench sediments (8,636 mbs) were incubated with phenol under 70 MPa for 23 days. The changes in phenol concentration and microbial biomass were both monitored throughout the incubation. As shown in Fig. 1, the phenol concentration of the sample without sediments had no change during the incubation, whereas the phenol concentration in the sample inoculated with sediments continued to decline from 0.70 to 0.61 mmol/L after 23 days. Simultaneously, the 16S rRNA gene copy numbers of bacteria significantly increased in the sediment-inoculated sample from 6.49 × 10^7^/g to 1.97 × 10^8^/g during the first 10 days, followed by a slight reduction to 1.17 × 10^8^/g for the rest of the incubation period. In contrast, the 16S rRNA gene copy numbers of archaea hardly changed during the 23-day incubation. These results indicate that phenol was degraded by active microbes from the hadal sediments rather than undergoing spontaneous decomposition in the simulated hadal environment. Furthermore, the results also suggest that active bacteria in the sediments were the primary phenol degraders, which showed significant increases in biomass during the high-pressure incubation. The slight reduction in biomass observed later in the incubation period may have been caused by insufficient oxygen to support the growth of all of the cells.

### Metatranscriptomic analysis identifies active community members and pathways putatively involved in phenol degradation

**Fig. 2:**
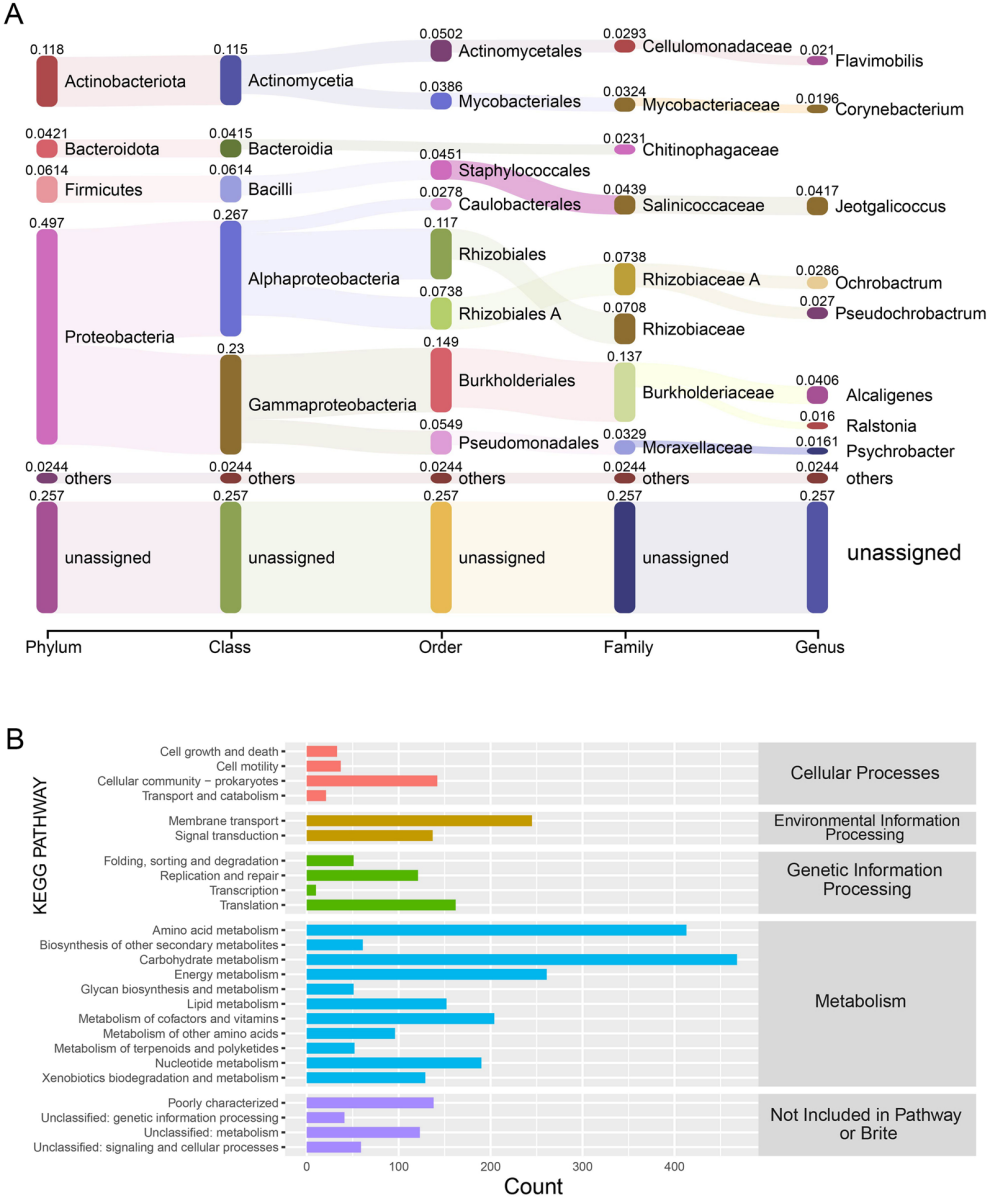
Metatranscriptomic analysis of the hadal-derived phenol degrading bacterial consortium under high-pressure incubation. A taxonomic compositions of the phylum, class, order, family, and genus levels based on the metatranscriptome. B Functional catalog annotations of the metatranscriptome based on KEGG pathway analysis

To gain insights into the active microbes and their active intracellular metabolism in trench sediments with phenolic compounds in the hadal environment, the metatranscriptome of microbes from Mariana Trench sediments after a 4-day incubation with phenol at 70 MPa was sequenced. After quality control of the 9.77 Gbp of raw data, 5.02 Gbp of clean reads were obtained. Of the total metatranscriptomic reads, 25.71% were unassigned hits, while bacterial transcripts constituted 71.85% of the total reads. The bacterial transcripts were dominated by the *Proteobacteria* phylum (49.70%), followed by *Actinobacteria* (11.80%), *Firmicutes* (6.14%), and *Bacteroidota* (4.21%) (Fig. 2A). *Alphaproteobacteria*, *Gammaproteobacteria*, *Actinomycetia*, *Bacilli*, and *Bacteroidia* were the main bacterial classes of the bacterial transcripts. At the order level, *Burkholderiales*, *Rhizobiales*, *Rhizobiales_A*, *Pseudomonadales*, *Actinomycetales*, *Staphylococcales*, *Mycobacteriales*, *Caulobacterales*, *Chitinophagales*, and *Sphingomonadales* were the top ten most abundant taxons transcribed under the extremely high-pressure conditions. In particular, at the family level *Burkholderiaceae* constituted up to 13.69% of the transcripts, of which *Alcaligenes,* at the genus level, contributed 4.06% of the transcripts.

After assembly of the clean reads, 55,065 contigs were obtained and subjected to subsequent analysis. Based on the functional annotations determined with the Kyoto Encyclopedia of Genes and Genomes (KEGG) dataset, the genes related to cellular processes, environmental information processing, genetic information processing, and metabolism were highly transcribed (Fig. 2B). Of the genes involved in cellular processes, most were related to cellular community processes, including biofilm formation, quorum sensing, and chemotaxis. The environmental information processes genes were mainly related to membrane transport and signal transduction. In addition, genes related to genetic information were mainly involved in DNA replication and repair and the translation process. In particular, genes encoding amino acid metabolism, carbohydrate metabolism, energy metabolism, lipid metabolism, cofactors and vitamin metabolism, nucleotide metabolism, and xenobiotics biodegradation were transcribed at high levels (Fig. 2B). These results imply the presence of a variety of intracellular metabolic activities in diverse microbes from trench sediments incubated with phenol under high pressure, simulating the hadal environment.

### Identification of phenol degradation pathways in metatranscriptomic dataset

**Fig. 3:**
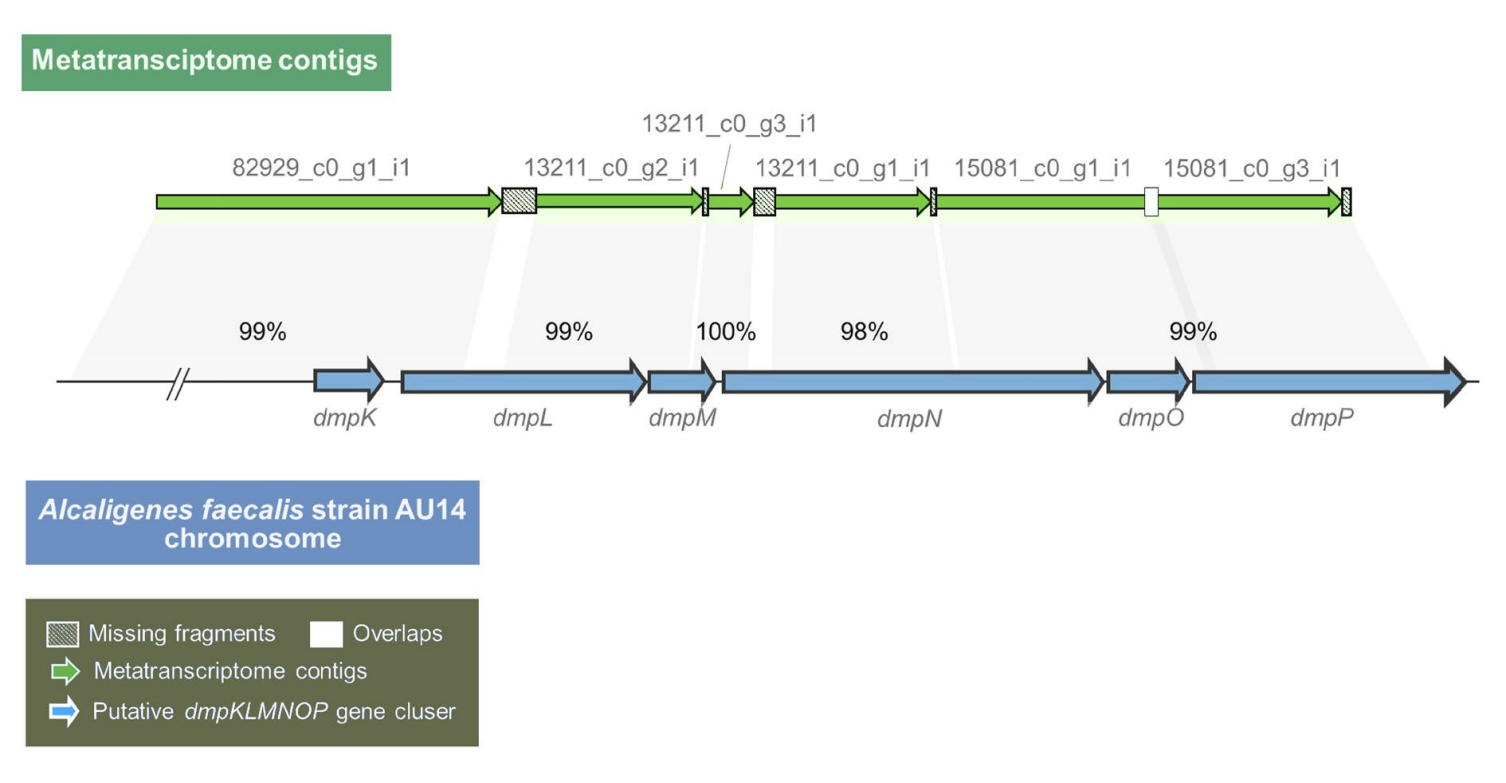
Six metatranscriptomic contigs arranged in accordance with the putative multicomponent phenol hydroxylase-encoding gene cluster from *Alcaligenes faecalis* strain AU14. The percentage indicates the nucleotide identities between the metatranscriptome contigs and the bacterial strain genome fragments

To identify the genes putatively involved in phenol degradation in the metatranscriptome, the amino acid sequences of functionally identified phenol catabolic enzymes were used as queries to search against the metatranscriptome using a local blast program. The amino acid sequences of two distinct phenol hydroxylases that catalyze the conversion of phenol to catechol, the multicomponent phenol hydroxylase DmpKLMNOP_*P*_ (accession numbers: P19729.1, P19730.1, P19731.1, P19732.1, P19733.1, and P19734.1 for each component) and the two-component phenol hydroxylase PheA1A2 (ABS30825.1 and ABS30826.1), were employed as queries. The former was sourced from the *Pseudomonas* sp. strain CF600 (Powlowski and Shingler 1994), while the latter was from *Rhodococcus erythropolis* CCM2595 (Zídková et al. 2013). As shown in Table 2, for each query, at least one hit was found. Although the putative *dmpKLMNOP* and *pheA1A2* genes were both transcribed in different species under high pressure of 70 MPa, the transcripts of *dmpKLMNOP* displayed high coverage and identities with the queries, and the transcripts of *pheA1A2* displayed low coverage with the queries in different contigs without overlap. Intriguingly, four transcribed contigs (82929_c0_g1_i1, 13211_c0_g2_i1, 13211_c0_g3_i1, and 15081_c0_g1_i1) were found to encode most of the units of the multicomponent phenol hydroxylase DmpKLMNOP_*P*_. This finding suggests that these four contigs comprised a polycistron. Subsequently, a long fragment (4954 bp) with five gaps, each less than 169 bp, was obtained by sequentially arranging the four contigs together with two other contigs (13211_c0_g1_i1 and 15081_c0_g3_i1). This fragment demonstrated excellent coverage of the putative multicomponent phenol hydroxylase-encoding genes in the *Alcaligenes faecalis* strain AU14 (CP031747.1) (Fig. 3) with high identities (from 98 to 100%) (Table 3). All of the above suggests that these six contigs were co-transcribed as a whole operon and encoded a putative multicomponent phenol hydroxylase during the high-pressure incubation with phenol.

**Table 2:** Metatranscriptome analysis of phenol degradation genes

Query sequence	Subject contig	Coverage (%)	Identity (%)	*E−*value	Genus	Contig abundance
DmpK (P19729.1)	82929_c0_g1_i1	88	66	1.00E−24	*Alcaligenes*	7.49889
DmpL (P19730.1)	53755_c0_g1_i1	31	61	5.00E−45	*Acinetobacter*	10.5075
	13211_c0_g2_i1	50	40	2.00E−44	*Alcaligenes*	8.10923
	82929_c0_g1_i1	21	61	4.00E−26	*Alcaligenes*	7.49889
DmpM (P19731.1)	13211_c0_g2_i1	59	53	6.00E−12	*Alcaligenes*	8.10923
	13211_c0_g3_i1	37	55	3.00E−08	*Alcaligenes*	44.2107
DmpN (P19732.1)	13211_c0_g1_i1	52	84	8.00E−172	*Alcaligenes*	5.26334
	83373_c0_g1_i1	28	74	2.00E−77	*Acinetobacter*	6.66122
	15081_c0_g1_i1	31	56	3.00E−61	*Alcaligenes*	8.85022
	13211_c0_g3_i1	7	68	4.00E−08	*Alcaligenes*	44.2107
DmpO (P19733.1)	15081_c0_g1_i1	99	38	1.00E−26	*Alcaligenes*	8.85022
DmpP (P19734.3)	15081_c0_g3_i1	90	66	2.00E−144	*Alcaligenes*	7.73262
	89313_c0_g1_i1	58	33	2.00E−23	*Brucella*	10.7983
	87366_c0_g1_i1	31	39	2.00E−24	*Tardiphaga*	7.07611
	51174_c0_g1_i1	62	28	8.00E−13	*Alcaligenes*	8.35832
	10285_c0_g1_i1	46	28	1.00E−16	*Alcaligenes*	10.3394
	908_c0_g1_i1	25	39	1.00E−13	*Caulobacter*	15.9503
	83040_c0_g1_i1	21	46	3.00E−13	*Shinella*	29.7874
	88943_c0_g1_i1	29	33	2.00E−12	*Alcaligenes*	22.9328
	15081_c0_g1_i1	10	67	1.00E−09	*Alcaligenes*	8.85022
	93443_c0_g1_i1	17	44	3.00E−10	*Alcaligenes*	5.00076
	30583_c0_g1_i1	19	38	4.00E−10	*uc_Alphaproteobacteria*	18.7068
	74025_c0_g1_i1	13	48	3.00E−09	*Achromobacter*	18.169
	4680_c0_g1_i1	20	40	7.00E−09	*Ralstonia*	55.5231
	88_c0_g1_i1	19	37	1.00E−08	*uc_Alphaproteobacteria*	6.15662
	85696_c0_g1_i1	35	33	3.00E−08	*Ralstonia*	13.8452
	371_c0_g2_i1	72	28	3.00E−07	*uc_Betaproteobacteria*	9.08508
	9649_c0_g2_i1	29	27	3.00E−07	*Alcaligenes*	35.9433
	7682_c0_g1_i1	21	34	6.00E−07		15.8679
	82147_c0_g1_i1	20	38	1.00E−06	*Corynebacterium*	5.15496
	89614_c0_g1_i1	19	36	4.00E−06	*Pandoraea*	19.9128
PheA1 (ABS30825.1)	92882_c0_g1_i1	17	54	2.00E−28	*Shinella*	15.3511
CatA (WP_096733829.1)	29188_c0_g2_i1	59	57	7.00E−74	*Alcaligenes*	16.4766
	88735_c0_g1_i1	48	41	2.00E−28	*Corynebacterium*	9.73894
	72333_c0_g1_i1	25	55	1.00E−15	*Rhizobiaceae*	12.8306
	73885_c0_g1_i1	37	31	7.00E−09	*Ralstonia*	35.4691
XylE (AAA23353.1)	–	–	–	–	–	–
CatE (NP_388705.2)	28148_c0_g2_i1	75	38	3.00E−43	*Micrococcus*	18.7056
	2566_c0_g2_i1	61	44	5.00E−41	*Brucellaceae*	8.69103
	95618_c0_g1_i1	52	46	1.00E−36	*Bacillales*	3.97318
	73750_c0_g1_i1	43	46	2.00E−29	*Bacillales*	8.22884
	4377_c0_g1_i1	36	44	3.00E−23	*Shinella*	17.8758
	38398_c0_g1_i1	43	41	2.00E−19	*Shinella*	17.1548
	74754_c0_g1_i1	31	44	3.00E−16	*Bradyrhizobiaceae*	31.4941
	14681_c0_g1_i1	85	26	3.00E−08	*Alcaligenes*	6.74765

**Table 3:** Nucleotide sequence identities between the putative *dmpKLMNOP* contigs from the metatranscriptome and the *Alcaligenes faecalis* strain AU14 chromosome

Contigs	*Alcaligenes faecalis* strain AU14 genome		Identities (%)
	Start	End	
82929_c0_g1_i1	2,459,259	2,461,031	99
13211_c0_g2_i1	2,461,200	2,461,986	99
13211_c0_g3_i1	2,461,996	2,462,224	100
13211_c0_g1_i1	2,462,312	2,463,116	98
15081_c0_g1_i1	2,463,139	2,464,178	99
15081_c0_g3_i1	2,464,163	2,464,115	99

**Fig. 4:**
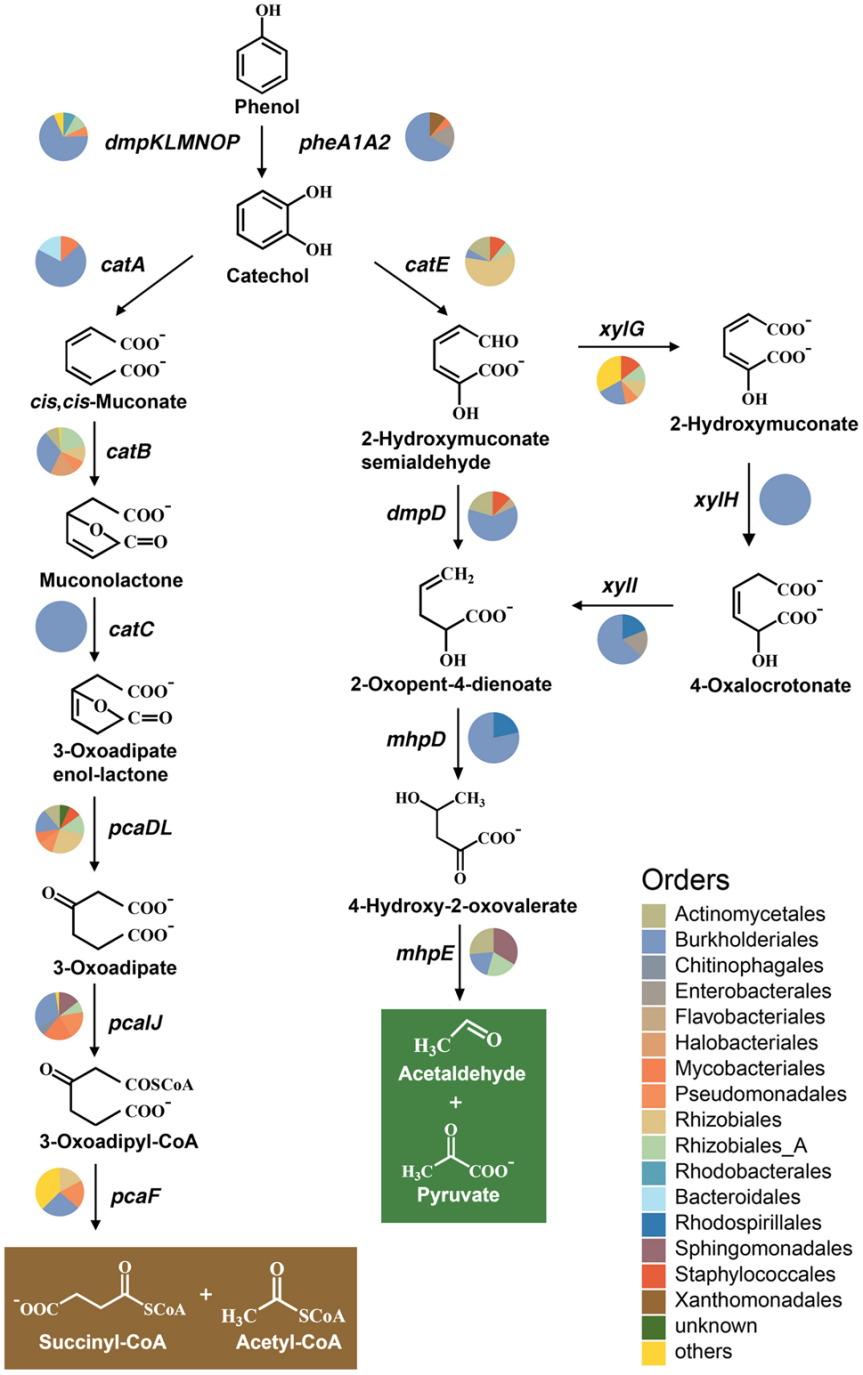
The deduced degradation pathway of phenol by the hadal trench-derived microbial consortium via *ortho*- and *meta*-cleavage pathways of catechol, based on the metatranscriptome under high pressure (70 MPa) incubation. Transcripts from different bacterial orders are depicted with pie charts for each gene

Next, genes involved in the following degradation of catechol via the *ortho*- or *meta*-cleavage pathway were also investigated in the metatranscriptome. The functionally identified enzymes CatA (catechol 1,2-dioxygenase, WP_096733829.1), as well its downstream enzymes (as shown in Table 2 and Supplementary Table S1) encoding the *ortho*-cleavage pathway, and CatE and XylE (catechol 2,3-dioxygenase, NP_388705.2 and AAA23353.1), as well its downstream enzymes (as shown in Table 2 and Supplementary Table S1) encoding the *meta*-cleavage pathway, were used as the queries. As shown in Fig. 4 and Table 2 and S1, all of the above enzyme-encoding genes, except for *xylE*, acquired at least one hit in the metatranscriptome, indicating that catechol, converted from phenol or other aromatics, could be further metabolized via the *ortho*- or *meta*-cleavage pathway, and enter the tricarboxylic acid cycle in trench-derived microbes at 70 MPa.

Taken together, the above findings indicate the presence of possible catabolic genes that encode complete phenol degradation via two types of catechol pathways in the metatranscriptome. The results also suggest that phenol or catechol utilizers were active in the Mariana Trench despite the high pressure.

### Trench-derived multicomponent hydroxylase catalyzes the hydroxylation of phenol and its methylated derivatives

A putative multicomponent phenol hydroxylase gene cluster, designated as *dmpKLMNOP*_*sed*_ (Fig. 3), was identified in the aforementioned long fragment that was assembled by sequentially arranging six metatranscriptome contigs. Then, the *dmp* gene cluster was synthesized de novo and expressed in *E. coli* BL21(DE3).

**Fig. 5:**
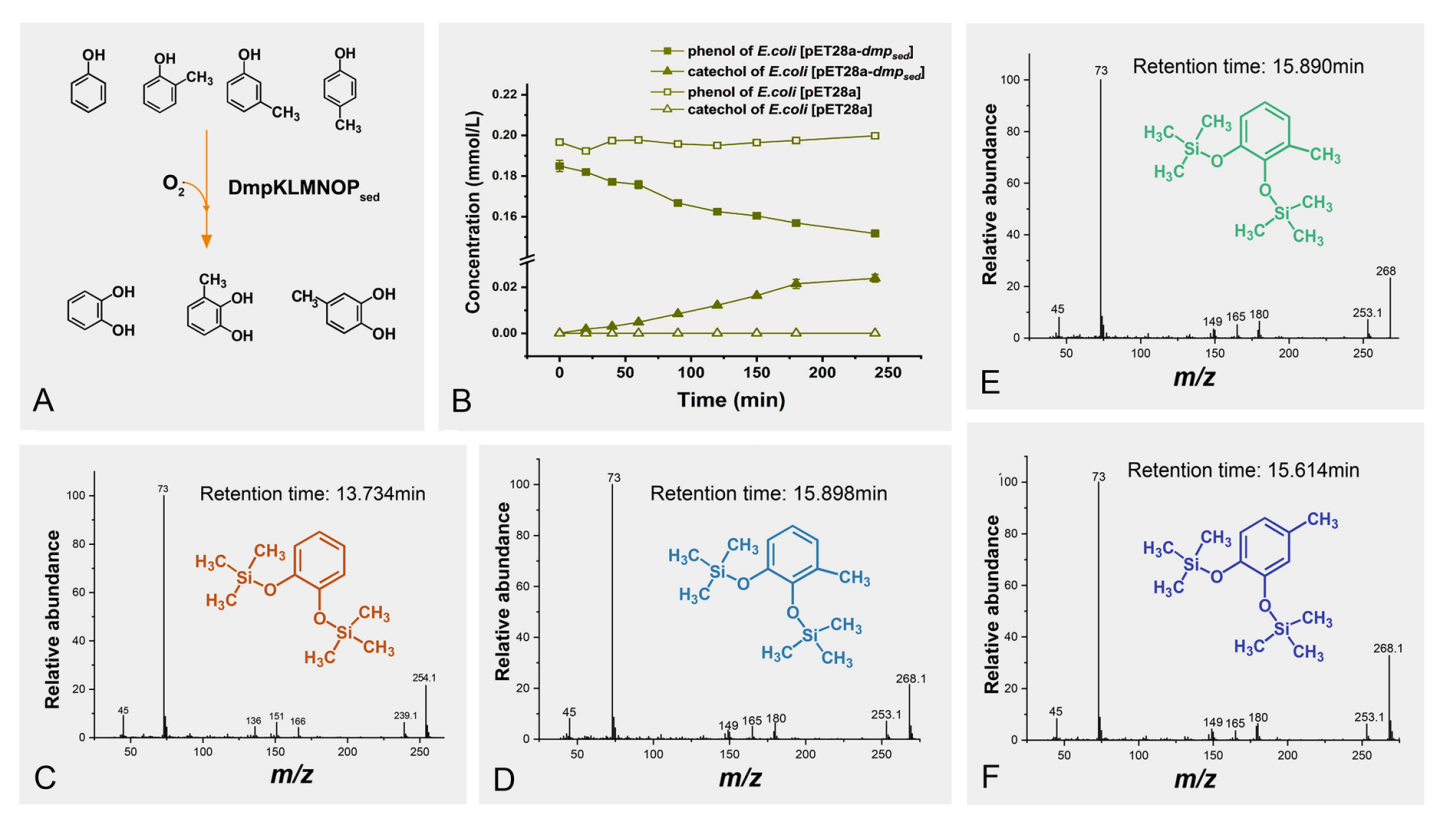
Biotransformation of phenol and cresols by *E. coli* BL21(DE3)[pET28a-*dmpKLMNOP*_*sed*_]. **A** The reaction scheme catalyzed by DmpKLMNOP_sed_ for phenol, *ortho*-cresol, *meta*-cresol, and *para*-cresol. **B** The time course of the decreasing phenol concentration and the catechol accumulation during a 4-h biotransformation. **C** GC–MS analysis of the trimethylsilyl (TMS) derivative of the phenol biotransformation product. **D** GC–MS analysis of the TMS derivative of the *ortho*-cresol biotransformation product. **E** GC–MS analysis of the TMS derivative of the *meta*-cresol biotransformation product. **F** GC–MS analysis of the TMS derivative of the *para*-cresol biotransformation product

To determine the function of DmpKLMNOP_sed_, biotransformation was performed on resting cells of strain BL21(DE3)[pET28a-*dmpKLMNOP*_*sed*_] with phenol and its three methylated derivatives (Fig. 5). High-performance liquid chromatography (HPLC) analyses showed that strain BL21(DE3)[pET28a-*dmpKLMNOP*_*sed*_] could transform phenol, whereas the control strain BL21(DE3)[pET28a] could not do so (Fig. 5B). Additionally, all of the *ortho*-, *meta*-, and *para*-cresols were also consumed by strain BL21(DE3)[pET28a-*dmpKLMNOP*_*sed*_] following the same trend. The resultant products with two hydroxyl groups derivatized with trimethylsilyl (TMS) were individually identified as catechol from phenol (Fig. 5C), 3-methylcatechol from *ortho*-cresol and *meta*-cresol (Fig. 5D, E), and 4-methylcatechol from *para*-cresol (Fig. 5F) by gas chromatography–mass spectrometry (GC–MS). These results also indicate the regioselectivity of the enzyme towards differentially substituted phenols, which is in accordance with previous reports (Hinteregger et al. 1992; Kukor and Olsen 1992). Additionally, the transformation rates of phenol and *ortho*-, *meta*-, and *para*-cresol were 0.253, 1.319, 0.529, and 0.185 μmol·L^−1^·min^−1^·per gram cells, respectively (Table 4). These results confirmed that the *dmpKLMNOP*_*sed*_ cluster, transcribed in the trench sediments enriched with phenol at 70 MPa, encoded a functional multicomponent phenol hydroxylase catalyzing the hydroxylation of phenol and its methylated derivatives (Fig. 5A).

**Table 4:** Specific activity of *E. coli* BL21(DE3)[pET28a-*dmpKLMNOP*_*sed*_] catalyzing the hydroxylation of phenol and its three methylated derivatives

Substrates	Specific activity (μmol·L^−1^·min^−1^·g^−1^)
Phenol	0.253
*ortho*-cresol	1.319
*meta*-cresol	0.529
*para*-cresol	0.185

### Abundance and diversity of key catabolic genes involved in the degradation of phenol and its derivatives in different hadal trenches

**Fig. 6:**
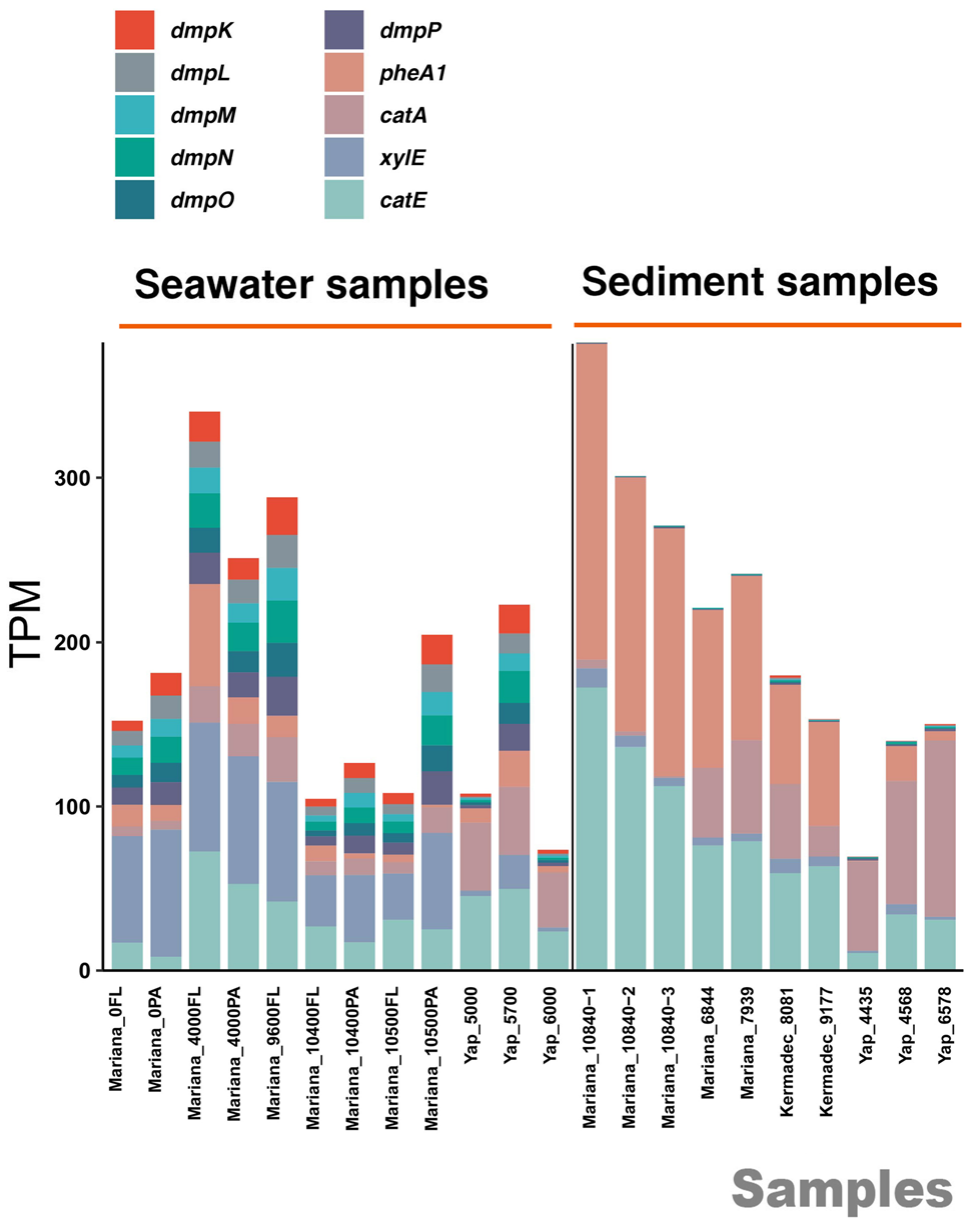
The abundance analysis of key genes involved in phenol hydroxylation and catechol cleavage within 22 publicly available hadal trench metagenomes from the Mariana Trench, Kermadec Trench, and Yap Trench. The transcripts per million (TPM) of different genes is indicated by the height of the bars in distinct colors

To analyze the abundance and diversity of genes encoding the initial catabolic enzymes (multicomponent phenol hydroxylase and two-component phenol hydroxylase) and the ring-cleavage enzymes (catechol 1,2-dioxygenase and catechol 2,3-dioxygenase) involved in the degradation of phenol and its derivatives in different hadal trenches, 22 publicly available metagenomes from the Mariana Trench (Chen et al. 2021; Liu et al. 2019), Kermadec Trench and Yap Trench (Zhang et al. 2018) were analyzed based on the KEGG ortholog assignments. The multicomponent phenol hydroxylase genes (*dmpKLMNOP*), the large subunit of the two-component phenol hydroxylase gene (*pheA1*), the catechol 1,2-dioxygenase gene (*catA*), and the catechol 2,3-dioxygenase genes (*xylE* and *catE*) were all present in metagenomes from these three hadal environments (Fig. 6). The small subunit of the two-component phenol hydroxylase gene (*pheA2*) was not analyzed due to the absence of information in KEGG. Specifically, all six of the *dmp* genes were more abundant in seawater samples than in sediment samples. Further, the relative abundance of *pheA1* was similar to that of each of the *dmp* genes in seawater samples but was more enriched in sediment samples. However, genes for catechol ring fission were more evenly dispersed among both seawater and sediment samples. Notably, *catA* was more abundant in Mariana Trench sediment samples, Kermadec Trench and Yap Trench samples, while *xylE* was more abundant in Mariana seawater samples.

**Fig. 7:**
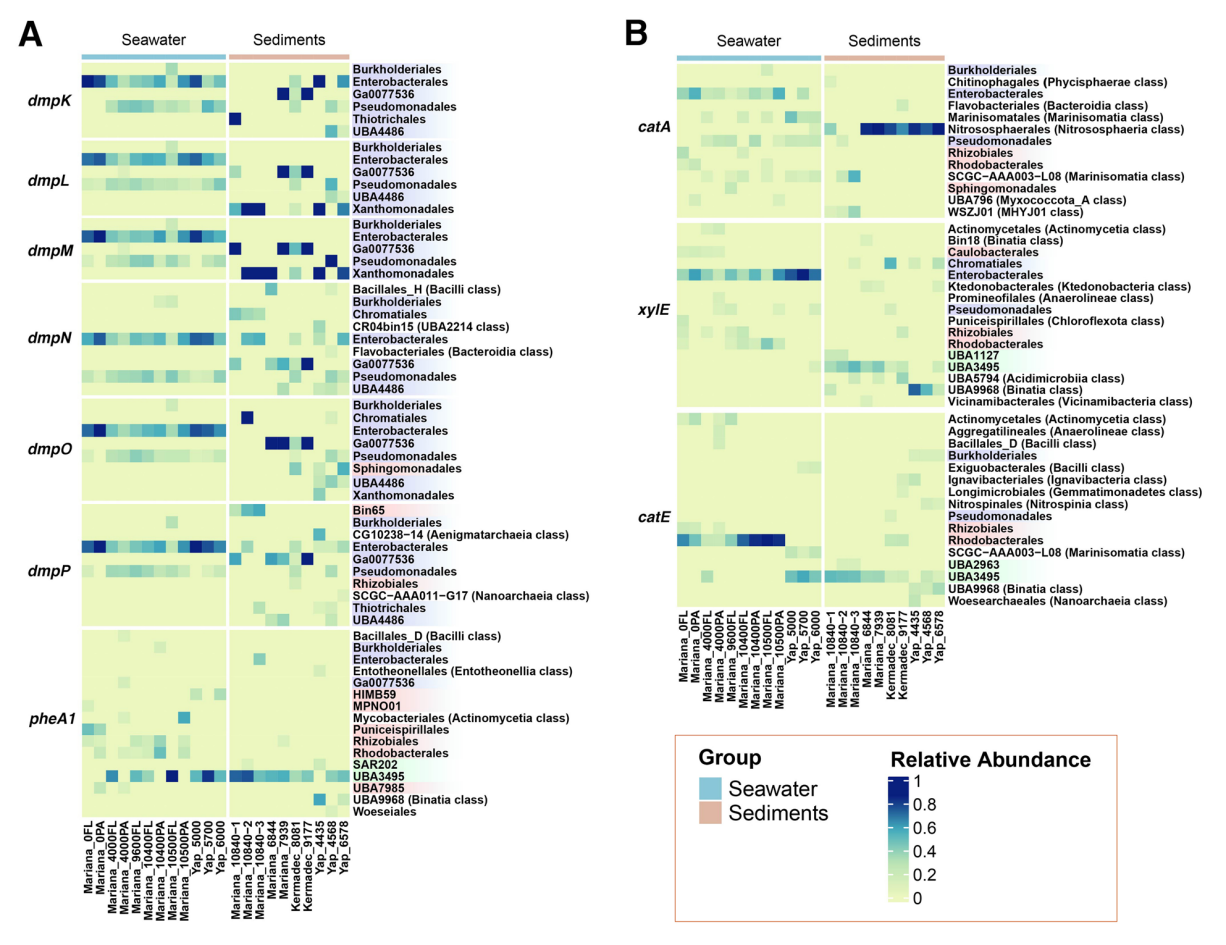
The taxonomic analysis of key genes involved in phenol hydroxylation and catechol cleavage within different hadal trench metagenomes. The relative abundance of different taxonomies is shown with a color gradient. Orders from *Gammaproteobacteria*, *Alphaproteobacteria,* and *Dehalococcoidia* classes are indicated in blue, red, and green, respectively. Orders from other classes are indicated in brackets. The samples were categorized into seawater and sediment groups, represented by light blue and pink bars, respectively

To further investigate the key microbial taxa responsible for phenol degradation in the different trench environments, taxonomic annotation was conducted on the putative key catabolic genes involved in trench-derived phenol degradation. The relative abundance of different microbial taxa at the order level was calculated for each gene across all of the trench samples. Taxa with a relative abundance above 0.1 were preserved and displayed. As shown in Fig. 7A, *Enterobacterales*, *Pseudomonadales*, and *Burkholderiales* comprised the three main taxa encoding *dmp* genes in seawater samples, which all belong to the *Gammaproteobacteria* class. In trench sediment samples, the *dmp* genes were predominantly present in orders that included *Enterobacterales*, *Ga0077536*, *Thiotrichales*, *UBA4486*, and *Xanthomonadales*. These results imply that these members of *Gammaproteobacteria* may play important roles in the phenol hydroxylation process in trench environments. Interestingly, there was a consistent distribution of *dmp* gene-harboring microorganisms from terrestrial environments, such as *Pseudomonas* sp. (Powlowski and Shingler 1994; Sazinsky et al. 2006) and *Acinetobacter* sp. (Doukyu et al. 2003), which also predominantly belong to *Gammaproteobacteria*, specifically within the orders *Pseudomonadales* and *Burkholderiales*. However, *UBA3495* from *Dehalococcoidia*, along with *Rhizobiales* and *Rhodobacterales* from *Alphaproteobacteria*, comprised the main orders encoding the *pheA1* gene in the three trenches, with the *Alphaproteobacteria* taxa more abundant in the Mariana seawater samples (Fig. 7A).

In contrast, the catechol ring-cleavage genes were encoded by a more diverse range of taxa (Fig. 7B). Not limited to *Gammaproteobacteria*, such as *Enterobacterales* and *Pseudomonadales*, *catA* was also found in several orders from *Alphaproteobacteria* and *Marinisomatia* in seawater samples. In sediment samples, *catA* was primarily present in the *Nitrososphaerales* order. Furthermore, *xylE* was also mainly found in *Enterobacterales* and several orders from *Alphaproteobacteria* in most of the trench seawater samples, whereas orders from *Dehalococcoidia*, *Binatia*, and *Acidimicrobiia* classes, and *Chromatiales* from the *Gammaproteobacteria* class were mostly encoded in trench sediment samples. In addition, *Rhodobacterales* and *Rhizobiales* from *Alphaproteobacteria* were the main taxa containing the *catE* gene within Mariana Trench seawater samples, with orders from *Dehalococcoidia*, *Binatia*, and *Marinisomatia* classes contributing more within Mariana Trench sediment samples and the other two trench environments.

In conclusion, in hadal seawater samples, *Gammaproteobacteria* and *Alphaproteobacteria* comprised the main potential phenol degraders. The members in *Gammaproteobacteria* primarily employed multicomponent phenol hydroxylase and two types of catechol dioxygenases, and the others in *Alphaproteobacteria* mainly harbored two-component phenol hydroxylase and catechol 2,3-dioxygenase. However, in hadal sediment samples, *Dehalococcoidia* potentially represented the most abundant phenol degraders, employing two-component phenol hydroxylase and catechol 2,3-dioxygenase.

### Phylogenetic analysis of major components of the multicomponent phenol hydroxylase from the hadal microbial metagenomes

**Fig. 8:**
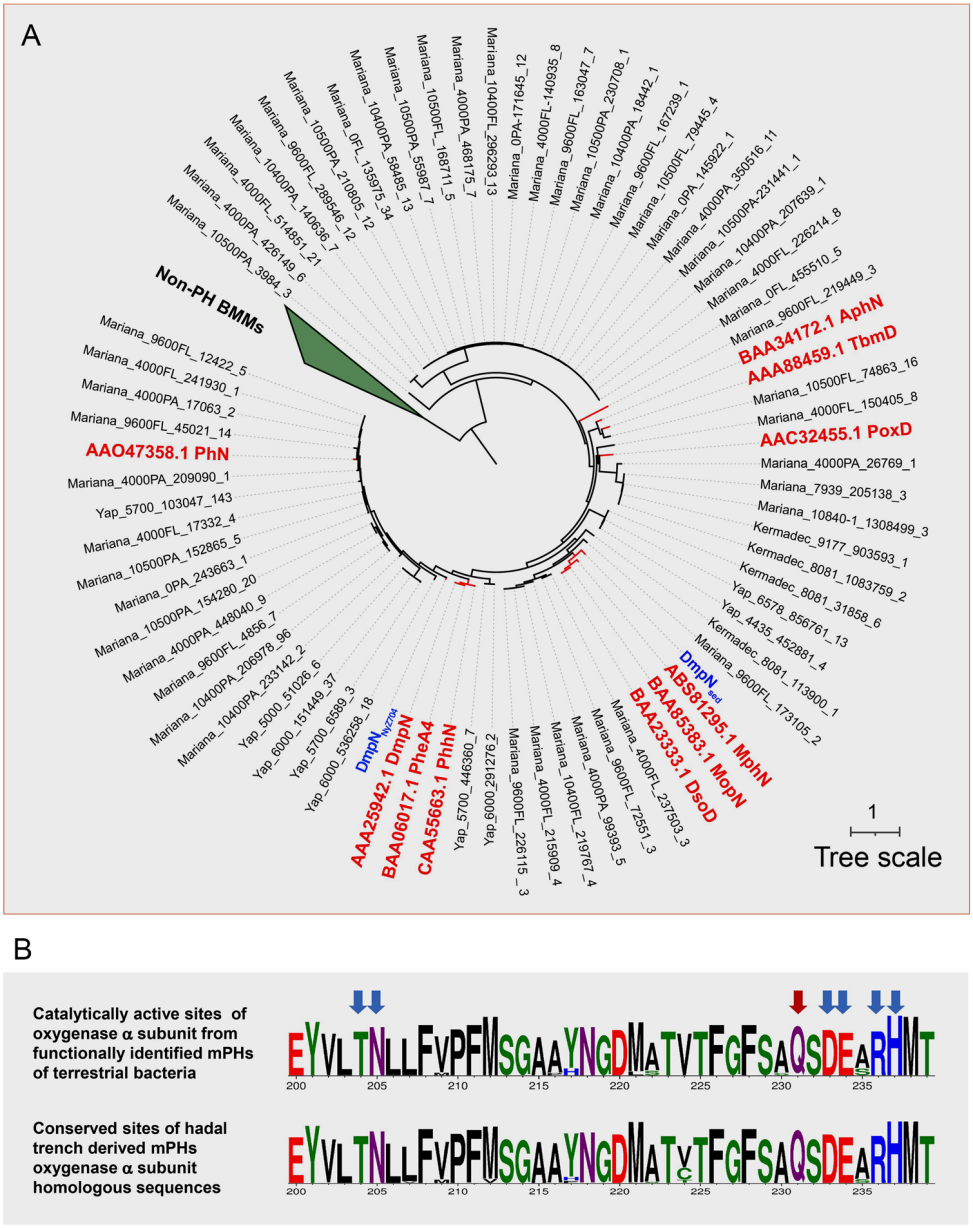
Phylogenetic analysis of the hadal trench-derived oxygenase component α subunit of the multicomponent phenol hydroxylase (mPHs). **A** A maximum-likelihood phylogenetic tree of the trench-derived sequences with the functionally identified bacterial multicomponent monooxygenases. The phylogenetic tree was constructed with 1,000 bootstrap replicates using iqtree, and visualized by iTOL (Letunic & Bork, 2019). Eight non-phenol hydroxylase bacterial multicomponent monooxygenases were utilized as outgroups, which are displayed in a collapsed clade. Ten sequences identified as phenol hydroxylases encoded by terrestrial bacteria and the sequence identified in this study are displayed in bold red font. Protein sequences that were functionally identified are presented with the corresponding UniProt entries. **B** Amino acid logo of the catalytically active site of the oxygenase α subunit from functionally identified sequences, and the putative catalytically active site of the oxygenase α subunit from publicly available hadal trench metagenomes. The arrow in red indicates the catalytically active site. The arrows in blue indicate the conserved sites involved in substrate entrance

In this study, it was found that multicomponent phenol hydroxylase-encoding genes (*dmpKLMNOP*) are widely distributed across global trench microbial metagenomes. To further investigate the evolution of hadal-derived multicomponent phenol hydroxylase, the sequences of the α-subunit of the oxygenase component from hadal microbial metagenomes (including DmpN_sed_ and DmpN_NyZ704_ from this study, and other hadal annotated proteins) were aligned with those of identified terrestrial enzymes. A maximum-likelihood phylogenetic tree was constructed based on the alignment. As shown in Fig. 8A, the sequences from hadal environments were closely clustered with the phenol hydroxylases encoded by terrestrial bacteria but separate from other non-phenol hydroxylase bacterial multicomponent monooxygenases. Additionally, the conserved amino acid analysis showed that all sequences from hadal microbial metagenomes shared highly conserved amino acid patterns with subunits of terrestrial multicomponent phenol hydroxylases in terms of key catalytically active sites (Fig. 8B).

### A culturable isolate capable of degrading phenol from Mariana Trench sediment

To isolate pure cultures of phenol degraders from the Mariana Trench sediments, two sediment samples (6,300 and 8636 mbs) were selected. A bacterium was obtained from the enrichment of sediments (6300 mbs) with phenol. The almost complete 16S rRNA gene exhibited a high identity (98.3%) to that of *Pseudomonas salina* strain XCD-X85 (NR137210.1) and this strain was designated *Pseudomonas* sp. strain NyZ704.

**Fig. 9:**
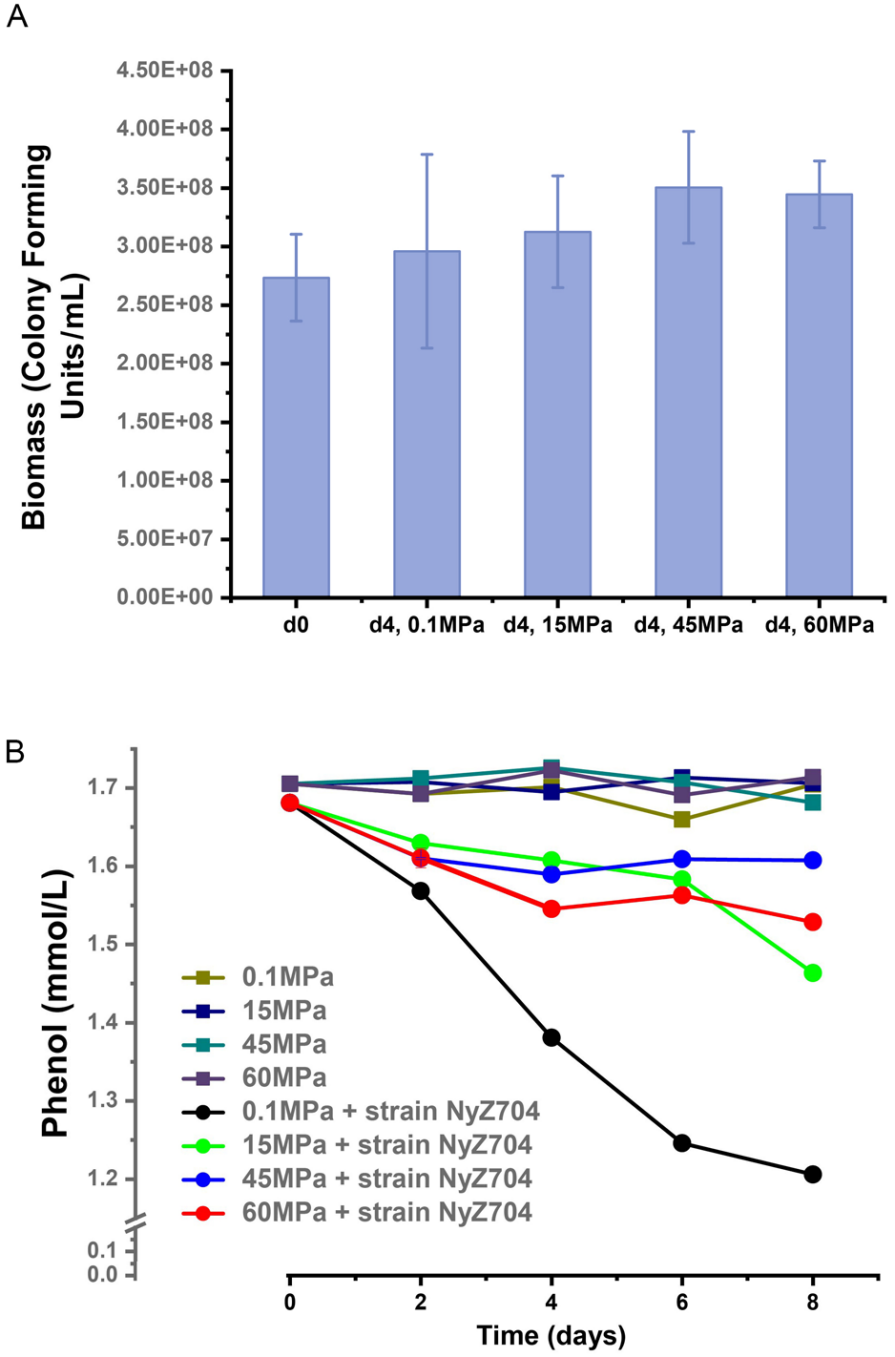
Mariana Trench-derived *Pseudomonas* sp. strain NyZ704 utilized phenol as the sole carbon source at both atmospheric pressure and high pressures. **A** Biomass accumulation of the strain NyZ704 at different pressures during a 4-day cultivation with phenol as the sole carbon source. **B** Phenol consumption at different pressures when cultivated with the strain NyZ704

Further, strain NyZ704 was incubated at 0.1 MPa (atmospheric pressure) and 15, 45, and 60 MPa (the simulated in situ hydrostatic pressures) with phenol as the sole carbon source. As shown in Fig. 9A, after a 4-day incubation, the biomass of strain NyZ704 (the initial biomass was 2.73 × 10^8^ colony forming units /mL) increased under all four hydrostatic pressures. During an 8-day incubation, phenol concentration also decreased under all conditions (Fig. 9B). In particular, the rate of decrease and the total amount of phenol consumed at 0.1 MPa were far higher than in the other three high pressure conditions (15, 45, and 60 MPa) (Fig. 9B). Further, the rate of decrease and the total reductions under either of the three high pressures were similar. The phenol concentration underwent no change at any of the three hydrostatic pressures (Fig. 9B) without inoculation. These findings clearly show that strain NyZ704 degraded phenol and utilized this phenolic compound for its growth under high hydrostatic pressure.

Notably, based on the analysis of the genome, strain NyZ704 contained catabolic genes encoding the complete phenol degradation pathway, and its encoded proteins individually showed high amino acid identities with those in the functionally identified terrestrial phenol degrader, *Pseudomonas* sp. strain CF600 (Table 5). In particular, in strain NyZ704, a putative *dmp* gene cluster encoded proteins that showed high identities of 68.29%, 72.64%, 76.67%, 83.73%, 58.62%, 77.62%, and 73.29% with the functionally identified multicomponent phenol hydroxylase DmpKLMNOP and catechol 2,3-dioxygenase DmpB in *Pseudomonas* sp. strain CF600 (Table 5).

**Table 5:** Annotation of gene clusters encoding the multicomponent phenol monooxygenase and catechol *meta*-cleavage pathway in *Pseudomonas* sp. strain NyZ704, and the alignment of amino acid sequences against each component of the phenol metabolic pathway in *Pseudomonas* sp. strain CF600

Gene list	Genetic location	Annotation	BlastP subject	BlastP identities (%)
*chr_2640*	2,885,453–2,885,644	4-oxalocrotonate tautomerase	DmpI	76.19
*chr_2641*	2,885,656–2,886,459	4-oxalocrotonate decarboxylase	DmpH	87.07
*chr_2642*	2,886,456–2,887,493	4-hyroxy-2-oxovalerate aldolase	DmpG	85.51
*chr_2643*	2,887,507–2,888,445	acetaldehyde dehydrogenase	DmpF	83.65
*chr_2644*	2,888,500–2,889,285	2-hydroxypent-2,4-dienoate hydratase	DmpE	86.92
*chr_2645*	2,889,288–2,890,154	2-hydroxymuconate semialdehyde hydrolase	DmpD	79.30
*chr_2646*	2,890,162–2,891,622	2-hydroxymuconic semialdehyde dehydrogenase	DmpC	84.57
*chr_2647*	2,891,654–2,892,574	Catechol 2,3-dioxygenase	DmpB	73.29
*chr_2648*	2,892,585–2,892,905	Ferrodoxin-like protein	DmpQ	64.77
*chr_2649*	2,893,022–2,894,083	Phenol hydroxylase component	DmpP	77.62
*chr_2650*	2,894,094–2,894,453	Phenol hydroxylase component	DmpO	58.82
*chr_2651*	2,894,529–2,896,076	Phenol hydroxylase component	DmpN	83.73
*chr_2652*	2,896,088–2,896,360	Phenol hydroxylase component	DmpM	76.67
*chr_2653*	2,896,357–2,897,358	Phenol hydroxylase component	DmpL	72.64
*chr_2654*	2,897,406–2,897,657	Phenol hydroxylase component	DmpK	68.29

Based on these findings, it can be inferred that the pure culture derived from Mariana Trench sediment exhibited activity that effectively degraded phenol through the catechol *meta*-cleavage pathway in hadal trenches. This observation aligns with the results obtained from the simulated high-pressure in situ transcriptome analysis conducted in this study.

## Discussion

Many aromatic compounds, especially phenolic compounds, are widely distributed throughout the ocean. The central role of microbes in carbon recycling from aromatic rings has been emphasized in several marine environments, including hydrothermal vents (Wang et al. 2011; Zhou et al. 2020), oil spills, and surface oceans (González-Gaya et al. 2019). In particular, the potential of indigenous hadal bacterial communities to metabolize aromatics was also suggested by cultivation-independent high-throughput sequencing in many studies (Chen et al. 2021; Liu et al. 2022; Wei et al. 2020; Xue et al. 2020; Zhang et al. 2018). Although some distinctive piezophilic and piezotolerant pure cultures have been isolated from hadal samples with nutrient-rich medium (Pathom-Aree et al. 2006b; Tamegai et al. 1997; Yang et al. 2020) and several were characterized as alkane utilizers (Liu et al. 2019), no consortium or pure culture has been reported to be able to degrade any type of aromatic compounds under atmospheric or high pressure conditions. Here, the utilization of phenol and its methylated derivatives (an important class of aromatic compounds) by cultivable hadal-derived bacteria with significant increase in biomass was demonstrated through high-pressure incubation at a maximum pressure of 70 MPa. In addition, *Pseudomonas* sp. strain NyZ704, a new bacterial isolate, was found to be capable of growing on phenol at a maximum pressure of 60 MPa (the simulated in situ hadal environment). This finding highlights the occurrence of active microorganisms in the hadal trench bottom and their capabilities of recycling carbon from relatively complex compounds containing aromatic rings under in situ high pressures.

Genes encoding several metabolic processes, including scattered putative simple aromatic catabolic genes, have been identified previously by metagenomic (Steiner et al. 2019) or metatranscriptomic analysis from hadal microbes (Gao et al. 2019). However, when aromatic compounds are used as carbon sources, their effects on microbial metabolism under high pressures are unknown. The transcribed genes involved in different metabolic processes herein were identified and quantified based on metatranscriptomic analysis from hadal microbial incubation with phenol as the sole carbon source under high pressure. Further, the active transcription of genes encoding membrane transport, membrane lipid biosynthesis, substrate utilization, cell motility, amino acid metabolism, and genetic information processing occurred during the high-pressure incubation with phenol in this study. The active expression of these microbial cellular functions was reported to be a common adaptation strategy adopted by piezophiles to cope with extreme pressures (Jebbar et al. 2015; Oger and Jebbar 2010). Hence, the hadal-derived microbial consortium capable of degrading phenol possibly adopted similar strategies to maintain cellular activities under an extreme environment.

Many terrestrial aerobic bacteria degrade phenolic compounds using catechol as the central intermediate via the β-ketoadipic acid pathway (Fuchs et al. 2011). In particular, the degradation of phenol and its derivatives was initiated by phenol hydroxylases, and the subsequent *ortho*- or *meta*-cleavage was catalyzed by catechol 1,2- or 2,3-dioxygenase before entering into the tricarboxylic acid cycle (Nesvera et al. 2015). In addition, phenol hydroxylases include three types, multicomponent phenol hydroxylase (DmpKLMNOP) (Shingler et al. 1992), two-component phenol hydroxylase (PheA1A2) (Zídková et al. 2013), and single-component phenol hydroxylase (Kukor and Olsen 1992). The first two phenol hydroxylases were more prevalent in known aerobic phenol degraders, while the single-component phenol hydroxylase was more of a promiscuous enzyme with a wide range of substrates (Hinteregger et al. 1992; Nesvera et al. 2015). Although phenolic compound degradation by terrestrial bacteria has been thoroughly identified at the molecular and biochemical levels, in hadal trenches, only the putative catechol 2,3-dioxygenase encoded gene has previously been identified in in situ transcription of the *Chloroflexi* clade SAR202 bacteria from the Mariana Trench (Wei et al. 2020). In this study, under high-pressure incubation with phenol, the transcripts of diverse catabolic genes encoding peripheral and central degradation of phenol via different pathways were revealed and quantitatively analyzed from Mariana Trench sediments. Notably, the multicomponent phenol hydroxylase-encoding gene cluster (*dmpKLMNOP*_*sed*_) not only was evidently transcribed in vivo, but was also active in vitro. This is an attempt to reveal the metabolic mechanism of aromatic compounds using phenol as a substrate in hadal trenches at the molecular and biochemical levels. In addition, due to mRNA degradation in prokaryotic microorganisms (Laalami et al. 2014), the lack of completeness of the two-component phenol hydroxylase transcripts and the absence of the single-component phenol hydroxylase transcripts cannot be ruled out as non-transcribed but instead may be the result of a short half-life of their mRNA.

Further, transcribed genes encoding various phenol hydroxylases, catechol 1,2-dioxygenase, and catechol 2,3-dioxygenase were all found to be widely distributed in different hadal metagenomics (22 trench samples from three different hadal trenches). In particular, *dmpKLMNOP* was found to be widely distributed in hadal trenches despite being found in other environments, including an aromatic compound-contaminated aquifer (Chen et al. 2022). The *dmpKLMNOP* gene cluster typically exhibited a higher abundance in seawater samples (Fig. 6), which was attributed to the divergence in oxygen availability between seawater and sediment samples. The dissolved oxygen concentration was relatively constant at 156–172 μmol/L at 4000 mbs in the hadal water column (about 40 MPa) (Nunoura et al. 2015), whereas benthic oxygen quickly decreased from concentrations higher than 170 μmol/L to approximately 50 μmol/L at 20 cm deep in the sediment (10,817 mbs, about 110 MPa) (Glud et al. 2013). Because phenol hydroxylation requires oxygen, the depletion of oxygen in hadal sediments may be a key factor limiting the abundance of *dmpKLMNOP* genes. Compared to phenol hydroxylase genes, catechol dioxygenases genes were also found in considerably high abundance within both trench water and sediments in this study, which was in accordance with previous studies on the vertical stratification of microbes in the deepest seawater column (Xue et al. 2020). Further, microbes living within sediments may possess other peripheral pathways that generate the central intermediate, contributing to the imbalance of *dmpKLMNOP* genes and catechol dioxygenase genes within the hadal sediment samples. Notably, the two-component phenol hydroxylase genes were shown to be of great abundance in sediment samples; however, more investigations are needed to confirm their actual function in catabolic processes.

### Supplementary Information

Below is the link to the electronic supplementary material.

Supplementary file1 (XLSX 29 KB)

## Data Availability

GenBank accession numbers for all of the mentioned genomes, proteins, genes, and publicly available metagenomes have been given in the text. The metatranscriptome and *Pseudomonas* sp. strain NyZ704 genome sequences have been deposited in GenBank under BioProject numbers PRJNA1020638 and PRJNA1020299, respectively.
